# Prodromal headache in anti‐NMDAR encephalitis: An epiphenomenon of NMDAR autoimmunity

**DOI:** 10.1002/brb3.1012

**Published:** 2018-06-01

**Authors:** Naomi Tominaga, Naomi Kanazawa, Atsushi Kaneko, Juntaro Kaneko, Eiji Kitamura, Hiroto Nakagawa, Kazutoshi Nishiyama, Takahiro Iizuka

**Affiliations:** ^1^ Department of Neurology Kitasato University School of Medicine Sagamihara Japan; ^2^ Department of Neurology Kagoshima City Medical Association Hospital Kagoshima Japan

**Keywords:** cerebrospinal fluid, critical care, epilepsy, headache, immunology, intensive care

## Abstract

**Objective:**

To investigate the nature of prodromal headache in anti‐NMDA receptor (NMDAR) encephalitis.

**Methods:**

Retrospective review of the clinical information of 39 patients with anti‐NMDAR encephalitis admitted between January 1999 and September 2017. Five patients with an atypical presentation were excluded. Thus, in 34 patients (median 27 years [range, 12–47 years]; 28 [82%] female), the clinical features were compared between patients who initially reported headache and those who did not report.

**Results:**

Twenty‐two patients (65%) reported headache either transiently (*n *=* *5) or continuously (*n *=* *17). Encephalitic symptoms (psychobehavioral memory alterations, seizure, dyskinesias, or altered level of consciousness) developed in 20 patients with median 5.5 days (range, 1‐29 days) after headache onset. In one patient, NMDAR antibodies were detected in CSF 3 days after headache onset. Patients with headache had more frequently fever (14/22 [64%] vs. 2/12 [17%] *p *=* *0.013) and higher CSF pleocytosis (median white blood cells 79/μl [range, 6‐311/μl] vs. 30/μl [range, 2‐69/μl], *p *=* *0.035) than those without headache, but there was no difference in gender, age at onset, seizure, migraine, CSF oligoclonal band detection, elevated IgG index, tumor association, or brain MRI abnormalities between them.

**Conclusions:**

Headache often developed with fever and pleocytosis, but it was rapidly replaced by psychiatric symptoms. Based on current knowledge on the antibody‐mediated mechanisms that cause a decrease of synaptic NMDAR through crosslinking and internalization leading to a state mimicking “dissociative anesthesia,” we speculated that prodromal headache is not likely caused by direct effect of the autoantibodies but rather meningeal inflammation (noninfectious aseptic meningitis) that occurs in parallel to intrathecal antibody synthesis as an epiphenomenon of NMDAR autoimmunity. Psychobehavioral alterations following headache is an important clue to the diagnosis.

## 1 INTRODUCTION

Anti‐*N*‐methyl‐d‐aspartate receptor (NMDAR) encephalitis is the most common autoimmune encephalitis caused by autoantibodies against the extracellular conformal epitope on the NR1 subunit of the NMDAR (Dalmau et al., 2007, 2008). Patients typically present with predictable multistage illness beginning with prodromal illness and followed by prominent psychiatric symptoms and seizures (Dalmau et al., 2007, 2008; Iizuka et al., 2008; Titulaer et al., 2013). Afterward, they rapidly fall into an unresponsive state characterized by profoundly decreased level of consciousness, central hypoventilation, intractable orofacial‐limb dyskinesias, seizures, and autonomic symptoms (Dalmau et al., 2007, 2008; Iizuka et al., 2008; Titulaer et al., 2013). Prodromal symptoms can be seen in 86% of patients in a large cohort (Dalmau et al., 2008) and have been regarded as a viral prodrome (Kayser & Dalmau, 2011); however, it remains controversial whether these symptoms, including headache and fever, are directly caused by viral infection or autoimmune mechanism. A few studies (Schankin et al., 2016) have addressed the nature of headache in this disorder.

In this study, we reviewed the clinical features of patients with anti‐NMDAR encephalitis and report potential mechanisms of prodromal headache in this disorder.

## 2 METHODS

### 2.1 Subjects

We first reviewed the clinical information of 39 patients with anti‐NMDAR encephalitis who were admitted to Kitasato University Hospital or other academic or referral hospitals between January 1, 1999, and September 10, 2017. The diagnosis was made based on the diagnostic criteria (Graus et al., 2016) for “definite anti‐NMDAR encephalitis”, and the presence of NR1‐IgG antibodies was confirmed using fresh serum/cerebrospinal fluid (CSF) samples in 33 patients who presented after 2007, and archived samples obtained at the symptoms onset and kept in frozen in 6 patients who presented before 2007. Five atypical patients (Kaneko et al., 2018), who presented with isolated epileptic seizure (*n *=* *2), demyelinating disorder without encephalitic symptoms (*n *=* *1), autoimmune post‐herpes simplex encephalitis (post‐HSE) (*n *=* *1), or overlapping encephalitis and demyelinating disorder (*n *=* *1), were excluded from the subjects. In accordance with, 34 patients with a typical spectrum of anti‐NMDAR encephalitis were included.

Clinical information on symptoms, neurological signs, CSF, electroencephalogram (EEG), MRI, and treatment, were obtained from the authors or referring physicians. A written or oral informed consent was obtained from all patients or their relatives for antibody assays.

### 2.2 Evaluation of clinical features

Encephalitic symptoms were defined as acute or subacute onset of brain dysfunction symptoms caused by encephalitis including psychobehavior and memory alterations, speech dysfunction, decreased level of consciousness, seizures, involuntary movements, autonomic symptoms, or changes in sleep or respiration. Prodromal symptoms were defined as those that developed before the onset of encephalitic symptoms. Thus, headache, fever, or other associated symptoms that developed before the onset of encephalitic symptoms were regarded as prodromal symptoms, but headache or fever following the onset of encephalitic symptoms was not included in prodromal symptoms.

Based on the presence of new onset of headache before development of psychiatric symptoms or altered level of consciousness, the subjects were divided into two groups: “patients with headache” who complained of headache (*n *=* *22) and “those without headache” who did not complain of headache (*n *=* *12).

The clinical information was obtained from the patients or their relatives on referral or admission. The clinical features including gender, age at onset, prodromal fever, seizures, comorbid migraine, mechanical ventilation support, MRI, EEG, CSF findings, and tumor association, were compared between patients with and without headache. The interval from headache onset to encephalitic symptoms onset was determined in 20 patients with headache; 2 patients, who complained of headache with fever after the onset of encephalitic symptoms (complex partial seizure), were not included.

### 2.3 Antibody assays

Autoantibodies were determined using rat brain immunohistochemistry and cell‐based assays (Dalmau et al., 2007) at the laboratory of Josep Dalmau (University of Barcelona). In all patients, autoantibodies against the NR1 subunit of the NMDAR were detected in both serum and CSF (*n *=* *31), CSF only (*n *=* *1, serum obtained 9 months after symptoms presentation was negative), and in serum (*n *=* *2, CSF was not available). All patients were also examined for autoantibodies against other neuronal cell surface and synaptic proteins including γ‐aminobutyric acid‐A receptor (GABAaR), *γ*‐aminobutyric acid‐B receptor (GABAbR), alpha‐amino‐3‐hydroxy‐5‐methyl‐4‐isoxazolepropionic acid receptor (AMPAR), metabotropic glutamate receptor 5 (mGluR5), dipeptidyl‐peptidase‐like protein‐6 (DPPX), contactin‐associated protein‐like 2 (CASPR2), and leucine‐rich glioma inactivated‐1 (LGI1) with technique previously reported (Boronat et al., 2013; Dalmau et al., 2007; Lai et al., 2009, 2010; Lancaster et al., 2010, 2011; Petit‐Pedrol et al., 2014).

### 2.4 Statistical analysis

The Fisher exact test was performed for comparison of categorical variables, and the Mann–Whitney test was used for continuous variables. The statistical significance was set at *p *≤* *0.05. We used JMP, version 11.2.0 (SAS Institute Inc.) for statistical analyses.

### 2.5 Ethics committee

This retrospective study conducted for investigation of prodromal headache in patients with anti‐NMDAR encephalitis was approved by Institutional Review Boards of Kitasato University (B17‐144).

## 3 RESULTS

### 3.1 Clinical features of anti‐NMDAR encephalitis

Twenty‐eight of 34 patients (82%) were female; the median age at symptoms onset was 27 years (range, 12–47 years). Twenty‐two patients (65%) reported headache before losing their ability to speak or development of decreased level of consciousness. Twenty‐eight patients (82%) presented with prodromal symptoms; headache (20/28, 71%), fever (14/28, 50%), nausea/vomiting (6/28), general malaise (5/28), or cold not otherwise specified (3/28). The other 6 (18%) began with acute onset of encephalic symptoms, such as psychosomatic symptoms (*n *=* *2), simple partial seizure (*n *=* *1), complex partial seizure (*n *=* *2), or progressive bulbar palsy (*n *=* *1) without apparent prodromal symptoms.

Detailed information about headache symptoms, such as its location, quality, severity, duration, or accompanying symptoms, were not available in all patients due to altered level of consciousness or prominent psychiatric symptoms when they were referred to our hospital. However, throbbing pain, nausea, or vomiting, were reported by patients. A headache or fever was initially regarded as nonspecific cold by referring physicians, patients, or their relatives, but 3 patients were initially diagnosed with possible viral meningitis before the development of encephalitic symptoms (*Patients 1, 2, and 3,* supporting information Data S1).

One female patient (#1) presented with acute onset of headache and fever without encephalitic symptoms, and underwent a lumbar puncture 4 days after the onset of headache, which revealed 139 white blood cells (WBCs)/μl (mononuclear cells 98%), leading to a hospitalization with possible viral meningitis; however, on the day of admission nocturnal psychiatric symptoms developed. Afterward, she rapidly fell into an unresponsive state with a typical spectrum of anti‐NMDAR encephalitis. NMDAR antibodies were detected in CSF obtained 7 days after headache onset (the initial CSF was not examined). Another female patient (#2) presented with bilateral throbbing headache and fever. Seven days later, she was admitted to another hospital with possible viral meningitis (CSF WBCs 186/μl [mononuclear cells 95%]). From the evening on the 6^th^ hospital day she became delirium state. NMDAR antibodies were detected in CSF obtained 15 days after headache onset (the initial CSF was not examined for antibodies). The other female patient (#3) presented with headache and fever. Two days later psychiatric symptoms rapidly developed. Next day she was admitted to a hospital. CSF examination revealed 146 WBCs/μl (mononuclear cells 97%). NMDAR antibodies were subsequently confirmed in the CSF obtained 3 days after headache onset.

Headache symptoms were divided into two types in terms of the mode of onset. One was a transient type (*n *=* *5); initial headache resolved spontaneously or after taking simple analgesics before development of severe encephalitic features, the other was a continuous type (17/22, 77%); headache persisted and progressively worsened with fever, and were subsequently rapidly replaced by prominent psychiatric symptoms, seizure, or altered mental status. Four of 34 patients (12%) had a past medical history of migraine.

**Figure 1:**
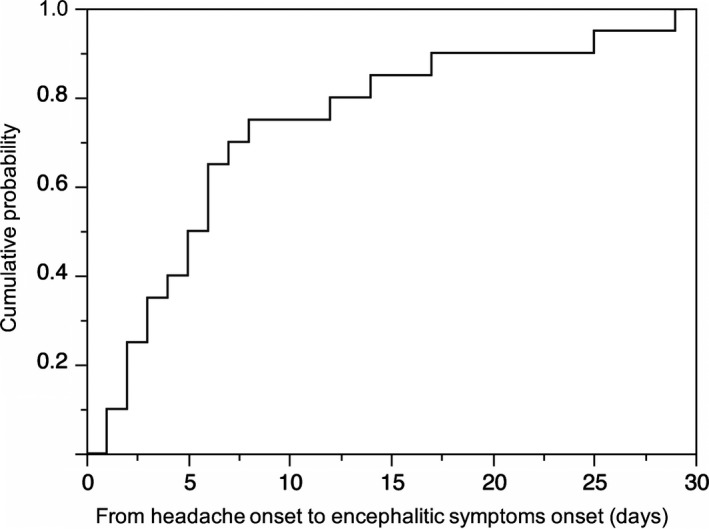
Cumulative probability of development of encephalitic symptoms after headache onset in patients with anti‐NMDA receptor encephalitis. This figure shows a cumulative probability of development of encephalitic symptoms after headache onset based on 20 patients who reported headache. Encephalitic symptoms developed within median 5.5 days (range, 1–29) of headache onset

Encephalitic symptoms included psychobehavioral or memory alterations (33/34, 97%), involuntary movements (32/34, 94%), seizure (30/34, 88%), status epilepticus (7/34, 21%) and altered level of consciousness (34/34, 100%). Mechanical ventilation support was required in 25 patients (74%). Tumor was found in 17 patients (50%): ovarian teratoma (*n *=* *16) or retroperitoneal germ cell tumor (*n *=* *1). These encephalitic symptoms developed within median 5.5 days (range, 1‐29) after headache onset in 20 patients; encephalitic symptoms developed in approximate 80% of patients within 14 days after headache onset (Figure 1).

### 3.2 Comparison of the clinical features in patients with and without headache

**Figure 2:**
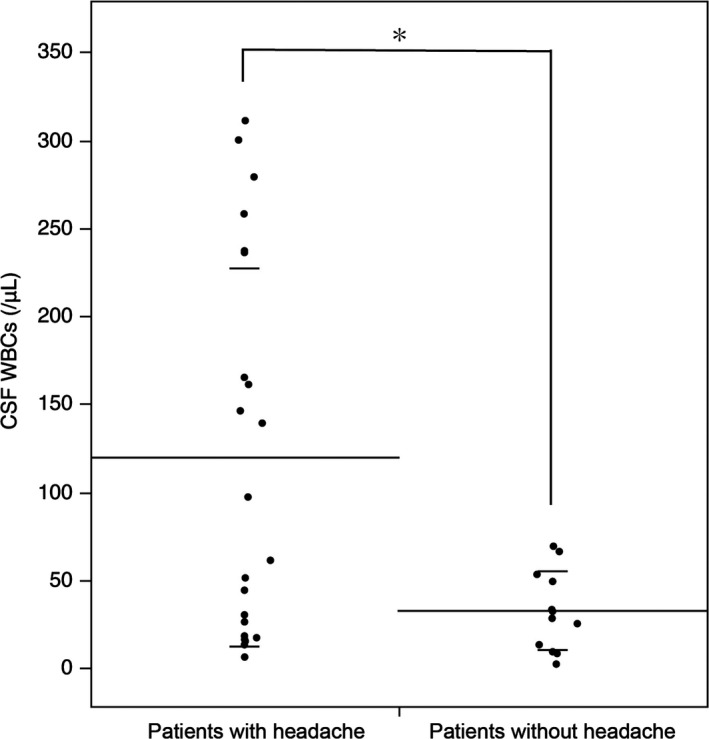
Comparison of CSF WBC counts between patients with and without headache in anti‐NMDA receptor encephalitis. The median white blood cell (WBC) counts in CSF are significantly higher in patients with headache than those without headache (79/μl [range, 6‐311/μl] vs. 30/μl [range, 2‐69/μl], *p *=* *0.035). However, six of 22 (27%) patients with headache had low CSF WBCs (<20/μl), while three of 12 (25%) patients without headache had CSF WBCs (>50/μl). Lines are shown as mean ± *SD*. *indicates significant difference between patients with and without headache

The clinical features of each group are shown in Table 1. Fever was more frequently seen in patients with headache than those without headache (14/22 [64%] vs. 2/12 [17%], *p *=* *0.013), but gender or median age at symptoms onset was not different. The median WBC counts in CSF at symptoms presentation were higher in patients with headache than those without headache (79/μl [range, 6‐311/μl] vs. 30/μl [range, 2‐69/μl], *p *=* *0.035). However, 6 of the 22 patients (27%) with headache had low CSF WBCs below 20/μl (range, 6‐18/μl), while 3 of the 12 patients (25%) without headache had relatively high CSF WBCs above 50/μl (range, 53‐69/μl) (Figure 2). In addition, 4 of the 6 patients (67%) with headache had fever despite low WBC counts, while 2 of the 3 patients (67%) without headache had no fever despite relative high WBC counts. The median CSF protein level, CSF oligoclonal bands detection rate, and frequency of elevated IgG index were higher in patients with headache than those without headache but not significantly different (Table 1). The frequency of mechanical ventilation support, brain MRI abnormalities at symptoms onset, EEG abnormalities, tumor, or comorbid migraine were not different between patients with and without headache.

**Table 1:** Comparison of clinical features between patients with and those without headache in anti‐NMDA receptor encephalitis

	Patients with headache (*n *=* *22)	Patients without headache (*n *=* *12)	*p* value
Gender female	17 (77%)	11 (92%)	0.389
Median age at symptoms onset (years)	27.0 (range, 12–47)	28.5 (range, 15–37)	0.718
Fever	14 (64%)	2 (17%)	0.013
Comorbid migraine	3 (14%)	1 (8%)	1.000
Seizures	21 (95%)	9 (75%)	0.115
Status epilepticus	4 (18%)	3 (25%)	0.677
Mechanical ventilation support	18 (82%)	7 (58%)	0.224
Brain MRI abnormalities at symptoms onset	8 (36%)	3 (25%)	0.705
EEG abnormalities^a^	19/21 (90%)	10/11 (91%)	1.000
CSF			
Median white blood cells (/μl)	79 (range, 6–311)	30 (range, 2–69)	0.035
Median protein (mg/dl)	36 (range, 14–220)	29 (range, 16–61)	0.220
Oligoclonal bands	10/17 (59%)	3/9 (33%)	0.411
Elevated IgG index (>0.73)	8/17 (47%)	1/4 (25%)	0.603
Tumors^b^	12 (55%)	5 (42%)	0.721

Notes. Patients with headache had more frequently fever and higher cerebrospinal fluid (CSF) pleocytosis than those without headache.

^a^EEG abnormalities include slowing and/or paroxysmal discharges; ^b^Tumors includes ovarian teratoma (*n *=* *16) and retroperitoneal germ cell tumor (*n *=* *1).

## 4 DISCUSSION

This study showed several important findings; (a) 65% of patients initially reported headache, (b) headache was often accompanied by fever but in most cases it was regarded as nonspecific cold, (c) headache developed either transiently or continuously but it was rapidly replaced by prominent psychiatric symptoms, (d) patients with headache had higher CSF pleocytosis than those without headache, and (e) NMDAR antibodies can be detected in CSF only 3 days after headache onset.

The previous study (Schankin et al., 2016) reported new‐onset headache to be a relevant symptom especially in patients with anti‐NMDAR encephalitis based on 40 patients with autoimmune encephalitis; however, only 16 (40%) had NMDAR antibodies. Only 9 of 40 patients (23%) reported new‐onset headache associated with encephalitis, 7 of whom had NMDAR antibodies. Relatively high detection rate of NMDAR antibodies among subjects with new‐onset headache may suggest some role of antibodies in headache, and glutamatergic dysfunction was presumed to be important for the generation of head pain, but no causative relationship of the antibodies to headache was reported (Schankin et al., 2016). While our study included 34 patients with anti‐NMDAR encephalitis, focusing on prodromal headache, a time interval from headache onset to encephalitic symptoms onset, and association between headache and various clinical parameters.

It remains unclear whether initial headache is directly caused by infection or autoimmune mechanism. Patients who developed anti‐NMDAR encephalitis following vaccination (Cartisano & Kicker, 2016; Hofmann, Baur, & Schroten, 2011) or herpes simplex encephalitis (HSE) (Armangue et al., 2015) have been reported, but viral infection has not been identified in most cases. In patients with post‐HSE (Armangue et al., 2015), the antibodies to the neuronal cell surface antigens (mostly NMDAR (Armangue, Martínez‐Hernández, Graus, & Dalmau, 2017)) have been detected a few weeks or months after the onset of HSE. In our case of post‐HSE, NMDAR antibodies were detected after at least 1 month after the onset of HSE as previously reported (Kaneko et al., 2018) Prodromal “viral‐like” disorder has been speculated to set off or enhance autoimmune response in patients with anti‐NMDAR encephalitis (Iizuka et al., 2008), but in our small series of patients 18% of those did not have prodromal symptoms, suggesting that this disorder could develop without apparent preceding infection or viral prodrome.

We found transient and continuous types of headache. The former headache resolved spontaneously before the development of encephalitic symptoms, whereas the latter headache persisted and worsened over a few days associated with fever; however, interestingly, most patients stopped complaining of headache in parallel with rapid development of psychiatric symptoms, and then they fell into a state of unresponsiveness (Iizuka et al., 2008). Such clinical worsening has been explained by antibody‐mediated decrease of NMDAR, which is directly correlated with the antibody titers (Dalmau, Lancaster, Martinez‐Hernandez, Rosenfeld, & Balice‐Gordon, 2011). Headache disappearance could be due to a rapid decline in consciousness, but loss of headache despite having the ability to complain of psychiatric symptoms is more likely due to an analgesic effect of the antibodies as discussed later.

The pathogenesis of prodromal headache in anti‐NMDAR encephalitis remains unclear. The NMDAR is expressed on both peripheral and central pain pathways including the trigeminal ganglion neurons and trigeminal nucleus caudalis in the brainstem, and the release of glutamate from the trigeminal nerve endings has been implicated in migraine headache (Kung et al., 2013; Xiao, Richter, & Hurley, 2008). However, antibody‐mediated activation of the pain pathway is unlikely because the NR1‐IgG antibodies functionally inhibit the NMDAR through crosslinking and internalization of the receptor (Dalmau et al., 2008, 2011). The clinical effect of NMDAR antibodies is thought to be similar to that of NMDAR antagonists, such as ketamine or phencyclidine (Dalmau et al., 2011; Lodge & Mercier, 2015), which cause a state called as “dissociative anesthesia”. Therefore, disappearance of headache rapidly replaced by psychiatric symptoms and subsequent development of the lack of withdrawal response to nociceptive stimuli despite eye open and their ability to hold‐up their arms (Iizuka et al., 2008) imply analgesic effect of the antibodies rather than pain‐inducing effect.

Headache is often accompanied by fever and CSF pleocytosis, thus headache is more likely due to meningeal inflammation and it can be classified into the category “7.3.2 Headache attributed to aseptic (noninfectious) meningitis” of the international classification of headache disorders (ICHD‐3) (Headache Classification Committee of the International Headache Society, 2018) Aseptic meningitis can occur in various systemic inflammatory disorders (Jarrin et al., 2016) including systemic lupus erythematosus, neurosarcoidosis, and neurobehçet’s disease. Thus, aseptic meningitis often seen in patients with anti‐NMDAR encephalitis could be immune‐mediated, and it may develop associated with ongoing intrathecal synthesis of NMDAR antibodies, when immune response is being activated, in which “meningeal inflammation” is a secondary phenomenon that occurs in parallel to “NMDAR autoimmunity” that is a primary phenomenon. Therefore, prodromal headache can be explained by an epiphenomenon of NMDAR autoimmunity, although we cannot exclude the possibility of infectious aseptic meningitis as a cause of prodromal headache.

It is of interest that 6 of 22 patients with headache had relatively low CSF WBC counts (<20/μl), while 3 of the 12 patients without headache had relatively high WBC counts (>50/μl). Although the threshold of headache may be different among patients, factors other than pleocytosis may also contribute to headache. Among those factors, high body temperature is an important factor because fever was more frequently seen in patients with headache than those without headache. Concomitant fever may decrease the threshold of pain, increasing the susceptibility to headache despite low CSF WBC counts. In fact, 4 of the 6 patients with headache had fever despite low CSF WBC counts, while 2 of the 3 patients without headache had no fever despite high CSF WBC counts.

Other possible factors involved in headache include increased intracranial pressure, inflammatory cortical lesions, individual susceptibility to headache (comorbid migraine), and elevated CSF inflammatory cytokines/chemokines. We did not find any case of elevated CSF opening pressure higher than 250 mmH_2_O but opening pressure was not available in all cases. Headache was not associated with brain MRI lesions at symptoms onset, gender, age at onset, or detection rate of oligoclonal bands or elevated IgG index. Migraine was found in 12% of patients, which is slightly higher than the prevalence of migraine in Japan (8.4%) (Sakai & Igarashi, 1997), but the frequency of migraine was not different between patients with and without headache in our series.

Although we did not measure CSF inflammatory cytokines/chemokines levels, it has been reported that interleukin‐6 (IL‐6), interleukin‐17 (IL‐17), and B‐cell‐attracting chemokine (C‐X‐C motif) ligand 13 (CXCL13) were elevated in CSF (Byun et al., 2016; Leypoldt et al., 2015; Liba et al., 2016). Both IL‐6 and IL‐17 are proinflammatory cytokines. High concentration of CSF level of CXCL13 was associated with the presence of prodromal fever or headache and intrathecal NMDAR‐antibody synthesis (Leypoldt et al., 2015). It is speculated that a potential inflammatory process (meningeal inflammation, viral, or yet unknown) could trigger a CXCL13‐mediated B‐cell attraction and contribute to the development of anti‐NMDAR encephalitis (Leypoldt et al., 2015); however, NMDAR antibodies were detected in CSF only 3 days after headache onset in our case. We speculate that intrathecal immune activation associated with NMDAR‐antibody synthesis may cause noninfectious aseptic meningitis in prodromal phase, resulting in headache and fever.

This study has limitations being a retrospective based on small numbers of patients with anti‐NMDAR encephalitis. Antibody titers were not determined in our cohort. No detailed clinical features of headache or its accompanying symptoms are available because most patients are unable to explain their symptoms on referral or admission due to rapid deterioration in consciousness level, coexistent psychiatric symptoms, or seizure. These patients are not able to recall prodromal symptoms due to retrograde amnesia even after recover of encephalitic symptoms. Thus, headache, its associated symptoms, and comorbid migraine may have been underdiagnosed due to coexistent psychiatric symptoms or memory loss. We cannot rule out such possibilities or any bias in history taking; however, we attempted to get information from patient’s relatives, referring physicians, or medical records as accurate as possible. There may be time lag between the onset headache or the peak of headache intensity and initial CSF examination; initial CSF findings may not reflect the severity of headache at onset.

Despite these limitations, this study demonstrated that prodromal headache often developed with fever, and it was rapidly replaced by psychiatric symptoms. Psychobehavioral alterations following headache is an important clue to the early diagnosis particularly in young woman who presented with new‐onset headache and fever without apparent cause.

## CONFLICT OF INTEREST

Kazutoshi Nishiyama received grants from Nippon Boehringer Ingelheim Co., Ltd., Daiichi Sankyo Co., Ltd., Astellas Pharma Inc., Otsuka Pharmaceutical Co., Ltd., Kyorin Pharmaceutical Co., Ltd., Dainippon Sumitomo Pharma Co., Ltd., Teijin Pharma Limited., Nihon Pharmaceutical Co., Ltd., Pfizer Inc., Bristol‐Myers Squibb, Japan Blood Products Organization, MSD, and Nihon Medi‐Physics Co., Ltd; Takahiro Iizuka is an editorial board member for Current Treatment Options in Neurology and Rinsho Shinkeigaku, and received a grant from Japan Epilepsy Research Foundation. Other authors have nothing to disclose regarding conflict of interests or commercial relationships including grants, honoraria, speaker’s lists, significant ownership, or financial support from pharmaceutical or other companies.

## AUTHOR CONTRIBUTION

NT, NK, and TI equally contributed to study concept and design, data acquisition, analysis and interpretation of the data, statistical analysis, and drafting and revising of the manuscript. AK, JK, EK, HN, and KN equally contributed to data acquisition and revising of the manuscript. All authors gave final approval of the version to be published.
