# Supplementary material for: Prodromal headache in anti‐NMDAR encephalitis: An epiphenomenon of NMDAR autoimmunity
Source: Brain Behav. 2018 Jun 1;8(7):e01012. doi: 10.1002/brb3.1012 (PMC6043713; doi:10.1002/brb3.1012)
Supplement: Supplementary file 1 [file BRB3-8-e01012-s001.docx]

**Supplementary Data**

This supplementary data provides additional information on 3 patients with anti-NMDAR encephalitis who presented with headache and were initially diagnosed with possible viral meningitis.

Abbreviations: FLAIR: fluid-attenuated inversion recovery; HSV-1: herpes simplex virus type 1; IVCPA: intravenous cyclophosphamide; IVIg: intravenous immunoglobulin; IVMP: intravenous high-dose methylprednisolone; mRS: modified Rankin Scale; *N*-methyl-D-aspartate: NMDA; NMDA receptor: NMDAR; mono: mononuclear cells; OCBs: oligoclonal bands; PLEX: plasma exchanges; WBCs: white blood cells

**Representative cases**

***Patient 1***

A 20-year-old Japanese woman presented with acute onset of occipital throbbing pain and vomiting in March 2009. Headache had gradually worsened, and 3 days later the temperature rose to 39°C. Next day, she presented to the emergency room at a city medical center. The temperature was 37.8℃. Headache intensity was aggravated by head shaking and physical activity but otherwise she was neurologically intact; she was alert, without psychiatric, behavioral or memory alterations, and her neck was supple.

The blood test-results were unremarkable, but initial CSF examination (*4 days after the onset of headache*) revealed 139 WBCs/μL (mono 98%), with elevated protein level (51 mg/dl) and normal glucose level (54 mg/dl). She was first diagnosed with possible viral meningitis. After admission she was treated with analgesics, but anxiety, loneliness, and insomnia developed at night on the day of admission, and nocturnal psychiatric symptoms recurred, but 2 days later she returned her home. However, hallucination and disorganized thinking developed at night. Next day (*7 days after the onset of headache*) she was re-admitted to the hospital.

Her past medical history was only remarkable for the left ovarian teratoma at 9 years of age; she was admitted to the hospital with abdominal pain due to a torsion of the left ovarian teratoma, and the tumor was removed and normal ovarian tissue was conserved.

On admission the temperature was 38.0℃. On neurologic examination she was awake but disoriented to time and place. CSF examination on admission revealed 117 WBCs/μL (mono 99%), normal protein level (30 mg/dl), and normal glucose level (62 mg/dl). PCR was negative for HSV-1. OCBs were not examined. A brain MRI on admission showed subtle increased FLAIR signal in the left insular cortex. She was treated with intravenous acyclovir (1500 mg/day) and IVMP (500 mg/day, 3 days) for presumed autoimmune encephalitis, and followed by oral prednisolone, but prominent psychiatric symptoms developed. On the 8^th^ hospital day generalized tonic seizure developed. After that, she repeatedly showed paroxysmal intermittent tonic posture extending her right upper extremity. On the 9^th^ hospital day, the right ovarian cyst was found on a pelvic MRI. Next day she was moved to an intensive care unit, intubated and sedated. She underwent a laparoscopic surgery; the right ovarian teratoma was removed while conserving the normal ovary, and irregular fatty tissue in the left ovary was removed. During her hospitalization the patient developed a typical spectrum of anti-NMDAR encephalitis, including intractable oro-facial limb dyskinesias, recurrent seizures, autonomic symptoms, prolonged decreased level of consciousness, and central hypoventilation requiring a 4-month mechanical ventilation support. She was treated with 2 cycles of IVMP (500-1000 mg/day, 3 days), and one cycle of IVIg (0.4 g/kg/day, 5 days), PLEX, and oral prednisolone (20 mg/day, gradually tapered off).

NMDAR-antibodies were confirmed in both serum and CSF obtained on the second admission (*7 days after the onset of headache*). Following the treatment with tumor resection, and first-line immunotherapy (IVMP, IVIg, and PLEX), all symptoms had gradually improved. One year later she returned to her university. The mRS at the last follow-up (7 years after presentation) was scored 0.

***Patient 2***

A 32-year-old Japanese woman had sore throat, and took analgesics, but general malaise persisted in August 2010. Four days later she began to complain of bilateral throbbing headache. Five days later the temperature rose to 38.0℃. Her headache persisted and worsened, and 7 days after the onset of headache she was admitted to another hospital with possible viral meningitis; CSF examination on admission revealed 186 WBCs/μL (mono 95%), elevated protein level (83 mg/dl), and normal glucose level (62 mg/dl). A brain MRI was normal. She was empirically treated with intravenous acyclovir (750 mg/day); however, high temperature and headache persisted. Follow-up CSF examination obtained on the 5^th^ hospital day revealed 236 WBCs/μL (mono 94%), elevated protein level (149 mg/dl), and normal glucose level (65 mg/dl). Next day (12 days after the onset of headache) psychiatric symptoms (hallucination) developed. She was treated with intravenous dexamethasone (7.6 mg/day), and followed by IVMP (1000 mg/day, 2 days); however, oral dyskinesias developed and high temperature persisted, and then she was transferred to our associated hospital for further evaluation. She had a past medical history of surgical resection of the left ovarian teratoma 10 years earlier (no detailed information was available).

On admission (day 1, 15 days after the onset of headache), the patient was awake but mute, and almost unresponsive to noxious stimuli or verbal commands. The cranial nerve function was normal, but she had perioral dyskinesias. Muscle tone was increased in the lower extremities. She had hyperreflexia, but no Babinski’s sign was seen. The neck was supple. The blood tests-results were unremarkable except mild leukocytosis. CSF examination on admission revealed 105 WBCs /μL (mono 98%), with elevated protein level (51 mg/dl). IgG index was elevated (1.12), but no OCB was examined. The CSF sample was reportedly positive for NMDAR-antibodies (*15 days after the onset of headache*).

On day 2, generalized tonic seizures developed. A pelvic CT demonstrated ovarian tumor in the right ovary. On day 3, she underwent surgical resection (pathologically confirmed as a mature teratoma). She began to receive continuous infusion of midazolam and propofol for persistent dyskinesias under mechanical ventilator support. She also received risperidone. On day 8, she received IVIg. On day 23, she began to receive phenytoin, carbamazepine, and intermittent administration of intravenous diazepam.

The subsequent clinical course was complicated by recurrent infections, sepsis, ileus, hyperammonemia, possible malignant hyperthermia, and drug allergy. Ten weeks after admission she was treated with IVMP (1000 mg/day, 3 days), followed by immunoadsorption, and the second course of IVIg, resulting in only partial improvement. However, 18 weeks after admission, involuntary movements exacerbated, for which she was started on PLEX and oral prednisolone (50 mg/day). The patient was treated with additional IVMP, and third course of IVIg. Twelve months after admission the patient was weaned off from a mechanical ventilator, but she remained in unresponsive wakefulness state for 20 months. Follow-up brain CT obtained 20 months after admission showed bilateral frontal lobe atrophy. The patient was then treated with monthly IVCPA (500 mg/m^2^, 6 cycles) from 21 months after admission resulting in remarkable improvement, and she was then transferred to a rehabilitation center 28 months after admission. The mRS at the last follow-up (74 months after presentation) was scored 0.

***Patient 3***

A 28-year-old Japanese woman presented with headache and fever in February 2017. Two days later, at 6 pm, she began to complain of difficulty talking and psychiatric symptoms. At 11 pm she presented to the emergency room at another hospital; she insisted on having flu but a rapid diagnostic test for influenza was negative, and she returned home. In the next morning, she looked dazed and repeated the same things to her husband; she could not recall anything that had happened the day before. After that she became incoherent. At 3 pm, she was brought to the emergency room at the other hospital. The temperature was 38.0°C. CSF examination (*3 days after the onset of headache)* revealed 146 WBCs/μL (mono 97%), normal protein level (27 mg/dl), and normal glucose level (67 mg/dl). A brain MRI was normal. She was admitted to the hospital with possible viral meningitis.

The patient was empirically treated with intravenous acyclovir, but generalized convulsive seizure developed on the day of admission. Intravenous levetiracetam was administered. On the 4^th^ hospital day, she began to receive IVMP (1000 mg/day, 3 days) but delirium and fever developed. Her subsequent clinical course was complicated by recurrent infections, recurrent seizure, fever, congestive heart failure, and acute renal failure associated with rhabdomyolysis (the highest serum CK level 162,768 U/L on the 14^th^ hospital day). On the 20^th^ hospital day a pelvic CT showed right ovarian tumor compatible with ovarian teratoma. On the 49^th^ hospital day, anemia had rapidly developed (Hb 4.2 g/dl). On the 52^nd^ hospital day, the patient was transferred to our hospital for further evaluation. She had a past medical history of menstrual migraine, and her family history was unremarkable.

On admission, the temperature was 39.3°C, and the oxygen saturation was 95% while she was breathing oxygen (3 L/minute) through a face mask. On examination she appeared pale, with marked pitting edema in her face and all extremities. A chest x-ray showed marked cardiomegaly with congestion. She was somnolent but able to follow simple commands. She had bulbar palsy, and severe muscle weakness; she was able to flex her elbow, wrist or fingers but not able to raise her upper or lower extremities. She had diffuse areflexia. Sensory examination was grossly intact. She had no headache on referral. The blood test-results showed severe anemia (Hb 4.5 g/dl), hypoalbuminemia (albumin 1.8 g/dl), kidney dysfunction, and elevated serum levels of CK (310 U/L), hepatic enzyme, CRP, and BNP (1487 pg/ml, normal < 18.4). CSF examination on admission reveal 3 WBCs/μL with normal level of protein (14 mg/dl) and glucose (74 mg/dl); PCR for HSV-1 was negative. OCBs were negative, and IgG index was not elevated. A whole body CT showed bilateral ovarian teratomas and severe pulmonary edema.

On the first day of admission the patient received red blood cell transfusion. Lamotrigine, carbamazepine, phenytoin, and valproate were discontinued. On the 4^th^ hospital day, she began to receive IVMP (1000 mg/day, 5 days) and IVIg (0.4g/kg/day, 5 days). On the 7^th^ hospital day, she was moved to ICU and intubated and sedated. On the 14^th^ hospital day she underwent tumor resections (pathologically confirmed as a mature teratoma in the right ovary and immature teratoma in the left). The clinical course was complicated by pulmonary embolism, anemia, severe thrombocytopenia, and recurrent infections. However, following the immunotherapies and supportive therapies, her condition had gradually improved. At 4 months after admission, the patients remained bedridden (the mRS scored 5), mainly due to disuse atrophy and pain associated with myositis ossificans, which developed in her pelvic girdle muscles probably caused by intramuscular hemorrhages, but cognitive function gradually improved. On day 141 the patient was transferred to a rehabilitation hospital. On discharge, the mRS was scored 4.

NMDAR-antibodies were detected in archived CSF obtained on admission to the referring hospital (*3 days after the onset of headache*) and in both serum and CSF obtained on transfer to our hospital 2 months after symptoms presentation.
